# Minimum Expected Delay-Based Routing Protocol (MEDR) for Delay Tolerant Mobile Sensor Networks

**DOI:** 10.3390/s100908348

**Published:** 2010-09-03

**Authors:** Yong Feng, Ming Liu, Xiaomin Wang, Haigang Gong

**Affiliations:** School of Computer Science and Engineering, University of Electronic Science and Technology of China, Chengdu 610054, China; E-Mails: fybraver@uestc.edu.cn (Y.F.); xiaomin.wang@126.com (X.M.W.); hggong@uestc.edu.cn (H.G.G.)

**Keywords:** delay tolerant mobile sensor networks, wireless sensor networks, routing protocol, minimum expected delay

## Abstract

It is a challenging work to develop efficient routing protocols for Delay Tolerant Mobile Sensor Networks (DTMSNs), which have several unique characteristics such as sensor mobility, intermittent connectivity, energy limit, and delay tolerability. In this paper, we propose a new routing protocol called Minimum Expected Delay-based Routing (MEDR) tailored for DTMSNs. MEDR achieves a good routing performance by finding and using the connected paths formed dynamically by mobile sensors. In MEDR, each sensor maintains two important parameters: Minimum Expected Delay (MED) and its expiration time. According to MED, messages will be delivered to the sensor that has at least a connected path with their hosting nodes, and has the shortest expected delay to communication directly with the sink node. Because of the changing network topology, the path is fragile and volatile, so we use the expiration time of MED to indicate the valid time of the path, and avoid wrong transmissions. Simulation results show that the proposed MEDR achieves a higher message delivery ratio with lower transmission overhead and data delivery delay than other DTMSN routing approaches.

## 1. Introduction

To deal with data gathering in mobile and extreme environments lacking continuous connectivity, Delay Tolerant Mobile Sensor Networks (DTMSNs) [1–5] have been proposed in recent years. DTMSNs belongs to the general category of Delay Tolerant Networks (DTNs) [6–12], occasionally connected networks that may suffer from frequent partitions. Although with similar hardware components, DTMSNs distinguish themselves from conventional sensor networks by some unique characteristics such as nodal mobility, intermittent connectivity, delay tolerability, limited battery supply and buffer and so on. A typical DTMSN consists of two types of nodes: the mobile sensor nodes and the sink nodes. The former, which can intermittently connect with each other, are attached to mobile objects for data gathering, and the latter are either placed at special locations or taken by some of mobile objects to collect data from sensors and forward them to the end user.

Obviously, it is difficult to form well connected end-to-end paths for mobile sensor nodes to transmit data to the sink nodes in DTMSNs, due to the sparse network density, short range radio and sensor node mobility, e.g., in scenarios like wildlife tracking for biological research, air quality monitoring, or flu virus tracking. Traditional data gathering approaches, which usually rely on a large number of densely deployed sensor nodes with short range radio to form a well connected end-to-end network, and collect the target data and transmit them to the sink nodes by collaborating together, cannot work effectively in DTMSNs. Therefore, how to develop efficient routing protocols, which can achieve high data delivery ratios with low transmission overhead and acceptable delay for DTMSNs, becomes the key issue.

Many existing works [13–18] cannot adapt to the characteristics of DTMSNs well. For example, due to too low data deliver ratios in direct transmission [13] and the tremendous amount of energy expense in epidemic algorithms [14], both basic routing schemes do not work efficiently in practical applications. Although mitigating the resource burden, MaxProp [15] and PREP [16], two variants of the epidemic protocol, still have very high transmission overhead. Later, RED [17] and FAD [18] consider the characteristics of DTMSN and make routing decisions based on historic records. They achieve better routing performance compared with other works, but the routing decision methods only depend on nodes’ utilities in one-hop scope, which overemphasizes the isolation and segmentation of networks, but don’t take the usual and local multiple-hop connected feature dynamically formed by moving nodes into account. Thus RED and FAD still have some drawbacks in routing performance.

**Figure 1.**
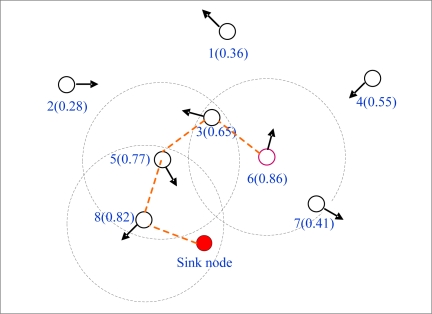
Illustration of next hop election.

For example, as shown in Figure 1, node 6 has two neighbors: nodes 3 and 7. According to the routing scheme based on the utility in RED or FAD, node 6 has to forward data messages to the nodes with higher delivery probability when it needs to send data messages to the sink node. For the delivery probability of node 6 is the highest among all its neighbors, therefore it cannot find the proper next hop to forward data, but there is evidently a multiple-hop connected path 6→3→5→8→sink on which node 6 could deliver data messages to the sink node. Here we note that in Figure 1, each dashed circle denotes the communication range of the node which is at the centre of the circle. Each broken line represents a link between two nodes. The number beside each node denotes the identity of the node, and the one in parentheses is used to indicate the delivery probability. The arrow on each node indicates the moving direction of the node.

As a result, we propose a new routing protocol called MEDR, which can efficiently find out and utilize temporary and local multiple-hop connected paths which are dynamically formed by moving nodes to improve the performance of data gathering. The major contributions of this work may be listed as follows:

♦ We introduce the concept of minimum expected delay (MED), which is employed to denote the expected earliest time that messages can be successfully delivered to the sink node.
♦ We propose the MEDR routing protocol for data gathering in DTMSNs with high data delivery ratio and low transmission overhead and delay.
♦ We compare the performance of the proposed protocol with several existing approaches and show that MEDR outperforms the existing approaches.

The rest of the paper is organized as follows: we review the related work in Section 2 and identify the problems in the existing works. We present the MEDR protocol in Section 3. The simulation is carried out, and the performance is evaluated in Section 4. Finally, we conclude this work in Section 5.

## 2. Related Work

Various approaches have been proposed to address the data gathering problem in DTMSNs, which aim to obtain high data delivery ratio at the cost of low transmission overhead and acceptable delivery delays. In [13], the authors presented a basic and simple routing protocol called direct transmission, where data is only allowed to be delivered when sensors are in direct proximity to the sinks. For messages are only sent directly from the source sensor node to the sink node, the protocol has relatively lower communication overhead but much longer delivery delay. Moreover, since it depends on the contacts of sensor nodes and the sink node, when there are very few sink nodes or the network is very sparse, the delivery ratio might be very low

Vahdat and Becker [14] propose an epidemic routing protocol to increase the data delivery ratio in partially connected networks. In epidemic routing scheme, two nodes exchange the data that they do not possess whenever they meet. Given unbounded bandwidth, buffer, and energy and so on, the extensive data exchanges ensure eventual message delivery at the cost of lots of redundant messages. However, the resources of bandwidth, buffer and energy are strictly limited in mobile sensor networks, which results in many messages dropped and poor performance in epidemic routing. Other examples of epidemic-based routing protocols include MaxProp [15] and PREP [16]. Although trying to mitigate the resource burden from flooding-based protocols, these two epidemic protocol variants still have very high transmission overhead, and thus may not be applicable for DTMSNs.

Wang and Wu [17] presented a replication-based efficient data delivery called RED, which consists of two components for data delivery and message management. First, data delivery uses a history-based method like ZebraNet to calculate the delivery probabilities of sensor nodes. Second, the message management algorithm decides the optimal erasure coding parameters based on sensor’s current delivery probability to improve the data delivery ratio. However, as indicated in [13], the optimization of erasure coding parameters is usually inaccurate, especially when the source is very far away from the sinks. In [18], Wang and Wu *et al.* also proposed a FAD protocol to increase the data delivery ratio in DTMSNs. Besides using the same delivery probability calculation method as RED, FAD further discusses how to constrain the number of data replications in the sensor network by using a fault tolerance value associated to each data message. However, that protocol still has a quite high transmission overhead.

The work by Juang *et al.* uses a history-based approach for routing in the ZebraNet project [19]. The routing decision here is made according to the past success rate with which each node transmits data packets to the sink nodes directly. However, the protocol may fail in delivering data messages generated by the sensor nodes that are far away from the sink nodes [20], so it is difficult for the simple scheme to reach good data delivery ratios. In [21], Small and Haas propose a system called SWIM to gather biological information about whales. In SWIM, data gathering is based on the assumption that sensor nodes move randomly and every node has the same chance to meet the sink. Thus each sensor node distributes a number of copies of a data packet to other nodes so as to reach the desired data delivery probability. However, in many practical applications different nodes may have different probabilities to reach the sink, so SWIM may not work efficiently.

Recently, several new routing protocols such as OPF [22], RCM [23] and EBR [24] have been proposed to achieve the desired performance. OPF assumes that all nodes have full routing information, that is, the mean inter-meeting times between all pairs of nodes. Though the authors discuss how to release the assumption from full routing information to partial routing information, the assumption is still strong, thus restricts the application range of OPF protocol. RCM presumes every node has cyclic motion pattern and uses a cyclic long-term metric to improve the routing performance. However, the assumption holds only in the kind of networks with periodic connectivity such as satellite communication, interplanetary communication, and social networks in which members are long-term and steady. EBR is an improved replication-based algorithm by making routing decision based on the rate of node encounters, and achieves good performance in the sceneries that the roles and activities of members are relatively fixed. However, EBR is not an ideal scheme for DTMSNs, due to its considerable energy consumption resulting from a large number of message copies.

## 3. Minimum Expected Delay Based Routing Protocol

Firstly, we assume initially that all the sensor nodes are randomly deployed in a square area of size M × M. The only static sink node is located at the center of the area. All the sensor nodes are homogeneous and have a unique ID number. The maximum transmission range of each node is fixed to *r*. Moreover, we further assume the mobile sensor network has the following characteristics: (a) The mobility of each sensor node in the given area is assumed to follow the RWP model; (b) The mobile sensor nodes in our model can easily obtain their locations from some attached extra device, for example GPS; (c) All nodes have their clock synchronized by using the NTP or the GPS clock itself [25].

Based on the assumptions above, we will present the methods to calculate the expiration time of the link which is formed between two nodes whose positions are in the communication scope of each other, as well as the two expected time values when a node meets and departs the sink node. After that, we present the calculation and update mechanism of nodes’ MED values. Lastly a detailed description of the MEDR routing algorithm is presented.

### 3.1. The Link Expiration Time

Based on our previous assumption, each mobile node can know its location coordination by GPS at any moment, and all the sensor nodes have synchronized clocks. Therefore, each mobile node can conveniently calculate its motion parameters (speed and direction), and broadcasts the parameters to its neighbors by the periodic hello messages. Assume two nodes *i* and *j* are within the transmission range *r* of each other at time *t*. Let the coordinates of *i* and *j* be (*x_i_,y_i_*) and (*x_j_,y_j_*), the speeds be *v_i_* and *v_j_*, and the moving directions be *θ_i_* and *θ_j_* (0 ≤ *θ_i_*, *θ_j_* ≤ 2*π*), respectively. According to the method in [26], we can calculate the link expiration time between node *i* and j, denoted as $T_{\mathrm{ij}}^{E}$:

(1) $$T_{\mathrm{ij}}^{E}=t+\frac{-(\mathrm{ab}+\mathrm{cd})+\sqrt{(a^{2}+c^{2})r^{2}-{\left(\mathrm{ad}-\mathrm{bc}\right)}^{2}}}{a^{2}+c^{2}}$$

where *a = v_i_* cos *θ_i_ − v_j_* cos *θ_j_, b = x_i_ − x_j_, c = v_i_* sin *θ_i_ − v_j_* sin *θ_j_, d = y_i_* *− y_j_*.

### 3.2. The Expected Time of Meeting and Departing the Sink Node

Let *O(x_o_,y_o_)* be the coordination of the sink node, then the communication range of the sink node is a circular region within the circle C ( *x*^2^ + *y*^2^ = *r*^2^ ). Let P(*x_i_,y_i_*) be the current location of node i, *v_i_* be the speed, and *θ_i_* (0 ≤ *θ_i_* ≤ 2*π*) be the moving direction. The process of calculating the expected time of node *i* meeting and departing the sink node, denoted as $T_{i}^{S}$ and $T_{i}^{E}$ respectively, can be categorized into the following three cases:

1. If the node *i* comes within the communication range of the sink node, then $T_{i}^{S}=t$, here *t* is the current time. The time that they will depart can be calculated out by Equation (1), that is, $T_{i}^{E}=T_{\mathrm{io}}^{E}$.
2. If the current moving path of the node *i*, *i.e.*, the ray L determined by P(*x_i_,y_i_*) and *θ_i_*, does not intersect the circle C (the communication range of the sink node), then $T_{i}^{S}=\infty$, $T_{i}^{E}=0$ since the node *i* will never meet the sink node in the near future.
3. If the above two cases cannot be held, then the ray L intersects with the communication range of the sink node. That means the node *i* is moving toward the sink node and will meet it with considerable probability. Let I_1_ and I_2_ be the two intersection points of the ray L and the circle C. Here, we ignore the instance that L tangents to C for the communication time between the two nodes is too short. Then:
  (2) $$T_{i}^{S}=t+PI_{1}/v_{i}$$
  (3) $$T_{i}^{E}=t+PI_{2}/v_{i}$$
  where PI_1_ and PI_2_ are the distance from P to I_1_ and I_2_ respectively, and PI_1_ < PI_2_.

### 3.3. The Minimum Expected Delay

In the paper, for any one node, e.g., node i, let MED_i_ denote the expected earliest time that messages forwarded by node *i* can be successfully delivered to the sink node, and $T_{i}^{M}$ be the period of validity of MED_i_. Initially, MED_i_ and $T_{i}^{M}$ depend on the expected time of node *i* itself meeting and departing the sink node. While receiving a hello message sent by one of its neighbors, e.g., node j, node *i* can get the mobility parameters and MED_j_ and $T_{j}^{M}$ of node *j* from the message, and calculate the link expiration time $T_{\mathrm{ij}}^{E}$. Node *i* maintains a neighbor list in which identity, mobility parameters, $T_{\mathrm{ij}}^{E}$, MED_j_ and $T_{j}^{M}$ for each neighbor *j* are recorded, and it periodically updates the list through adding new neighbors and removing nodes whose links already expired. According to the information in the neighbor list as well as the expected time of meeting and departing the sink node, node *i* periodically calculates MED_i_ and $T_{i}^{M}$ and announces them to all its neighbors through hello messages.

Let the current time be t, then for node *i* the process of calculating MED_i_ and $T_{i}^{M}$ is as follows:

1. If the node *i* comes within the communication range of the sink node, then MED_i_ = t, $T_{i}^{M}=T_{i}^{E}$. The calculation finishes. Otherwise, if node *i* has no any neighbor then goes to step (2), or else to step (3).
2. If $t<T_{i}^{E}$, then ${\text{MED}}_{i}=T_{i}^{S}$, $T_{i}^{M}=T_{i}^{E}$; or else MED_i_=∞, $T_{i}^{M}=0$. The calculation completes.
3. Let the neighbor set of node *i* be ∑ = {Ψ*_z_* |1≤ *z* ≤ *Z*}, here Z be the total number of neighbors of node i. Firstly *i* looks for whether at least a neighbor satisfying $T_{i\Psi_{z}}^{E}>t$, $T_{\Psi_{z}}^{M}>0$ in its neighbor set ∑ exists. If doesn’t exist, then going to step (2); otherwise, node *i* finds out such node(s) with the minimum MED value in all the neighbors satisfying $T_{i\Psi_{z}}^{E}>t$, $T_{\Psi_{z}}^{M}>0$. If the result is only one, then it is denoted as m; or else chooses the neighbor with the maximum value of $\text{min}\left(T_{\Psi_{z}}^{M},T_{i\Psi_{z}}^{E}\right)$ among the findings, denoted as m. If $t<T_{i}^{E}$ and $T_{t}^{S}\leq MED_{m}+t_{\mathrm{hop}}$, then ${\mathrm{MED}}_{i}=T_{i}^{S}$, $T_{i}^{M}=T_{i}^{E}$; otherwise *MED_i_* = *MED_m_* + *t_hop_*, $T_{i}^{M}=\text{min}(T_{m}^{M},T_{\mathrm{im}}^{E})-t_{\mathrm{hop}}$. Here *t_hop_* is a constant employed to indicate the estimated amount of time that a message is forwarded by a node to one of its neighbor(s), which includes the time required for the node to check its neighbor list and identify the next hop, and the propagation delay to transmit the message and so on. The impact of the size of *t_hop_* on performance will be discussed in Section 4 in detail.

**Figure 2.**
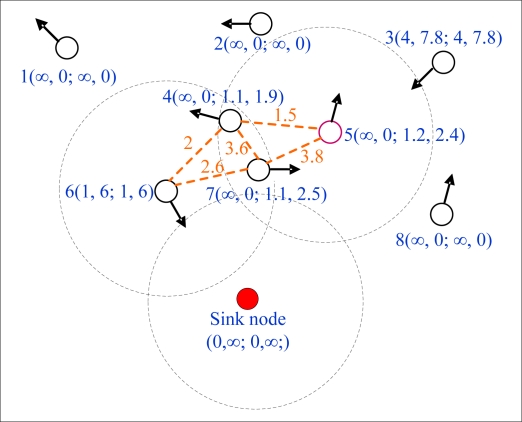
Illustration of computing nodes’ MED and expiration time.

Figure 2 shows the process of calculating MED and its valid period when t = 0 and *t_hop_* = 0.1 s. Here we note that each broken line represents a link between two nodes, and the number tagged on the broken line is the expiration time of the link. There is a pair of parentheses beside each node, in which the four numbers denote the expected time of meeting and departing the sink node, as well as MED and its valid period, respectively. As shown in Figure 2, the expected time of meeting with the sink node of node 6 is the minimum among all nodes, so ${\text{MED}}_{6}=T_{6}^{S}=1$, $T_{6}^{M}=T_{6}^{E}=6$. Through receiving hello message sent by node 6, node 4 and 7 update their MED and valid period as follows: MED_4_ = 1.1, $T_{4}^{M}=1.9$; MED_7_ = 1.1, $T_{7}^{M}=2.5$. Due to node 4 and 7 are also the neighbors of node 5, and MED_4_ is equivalent to MED_7_ but $\text{min}(T_{4}^{M},T_{4,5}^{E})=1.5$ is less than $\text{min}(T_{7}^{M},T_{7,5}^{E})=2.5$, therefore MED_5_ = 1.2, $T_{5}^{M}=2.4$. As to the other nodes (*i.e.*, node 1, 2, 3 and 8), the MED and valid period of each node completely lie on the expected time of meeting and departing the sink node because of having no any neighbor at the current time.

### 3.4. The Update Scheme of MED

With a view to saving energy, the period of sending hello messages for each node should not be too short, so this may make the performance of MEDR descend a little to update MED and its valid period only through hello messages, when the topology of the network changes frequently and rapidly. The reason is that the spread of MED between neighbors is not timely enough so that MEDR cannot find some paths that should be used for messages transmission. Therefore we introduce the update scheme of MED as follows:

For any one node (e.g., node i), while receiving a hello message sent by one of its neighbors (e.g., j), node *i* judges whether all the following three conditions hold: (a) node *j* is a new neighbor, *i.e.*, the neighbor list has no record about node j; (b) MED_j_ is valid and *MED_j_* + *t_hop_* < MED_i_; (c) node *i* has other neighbor except node j. If all of the three conditions hold, then node *i* updates its neighbor list, generates an update message and broadcasts the message to its neighbors. Or else, node *i* only updates its neighbor list. Here, update messages have the same content as hello messages, except the message type and the sending occasion.

Upon receiving an update message from a neighbor (e.g., node k), node *i* checks whether the following two conditions hold at the same time: (a) MED_k_ is valid and *MED_k_* + *t_hop_* < MED_i_; (b) node *i* has other neighbor except node k. If both of the two conditions above hold, then node *i* updates its neighbor list and broadcasts the update message to its neighbors; otherwise, node *i* only updates its neighbor list.

The update scheme of MED will increase the transmission overhead of the DTMSN to a certain extent. Fortunately, the overhead resulted from the update scheme is very limited, since the generation and transmitting of update messages strictly bounded by the aforementioned conditions. In the simulation experiments, the ratio of the total number of update messages to that of hello messages is only 4.6% in the worst case (*i.e.*, 180 mobile nodes deployed in the scenery of 200 × 200 m^2^) that the node density is the highest and the number of update messages is the most among all simulation scenarios. Therefore, the transmission overhead of the update scheme of MED is low and acceptable.

### 3.5. Data Transmission Algorithm

**Figure 3.**
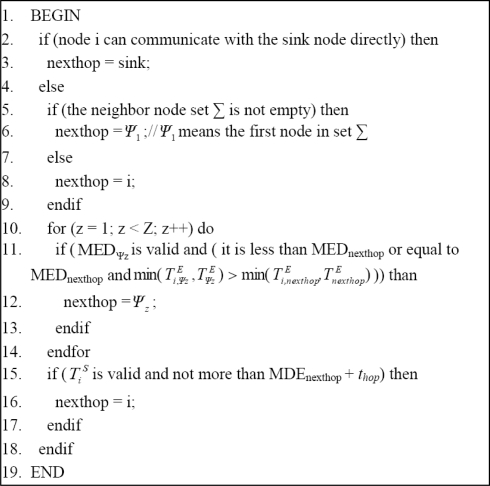
Pseudo-code of the routing algorithm.

In the MEDR algorithm, routing decision is made based on MED and its valid period. For any one node e.g., node i, let it have Z neighbors at the current time t and ∑ = {Ψ*_z_* | 1 ≤ *z* ≤ *Z*} be the set consisting of the Z neighbors. Through hello messages, node *i* learns the mobility parameters, MED and valid period of each neighbor, and further calculates out the link expiration time between it and every neighbor. When node *i* has a message M it needs to forward, the routing decision process is as follows: firstly node *i* finds out such node(s) with the minimum MED value in all neighbors satisfying $T_{i\Psi_{z}}^{E}>t$, $T_{\Psi_{z}}^{M}>t$. If it finds none, then node *i* has no proper next hop and the routing algorithm ends. Otherwise, if there is only one such neighbor, then it is denoted as m; or else node *i* chooses the neighbor with the maximum value of $\text{min}(T_{\Psi_{z}}^{M},T_{i\Psi_{z}}^{E})$ among the findings, denoted as m. Secondly, node *i* checks whether $T_{i}^{E}>t$ and $T_{i}^{S}\leq {\mathrm{MED}}_{m}+t_{\mathrm{hop}}$ both hold. If so, then node m is not the proper next hop and the routing algorithm ends; otherwise node m is just the next hop what node *i* is looking for, and thus message M will be forwarded to it. The pseudo-code of the routing algorithm is shown in Figure 3.

## 4. Simulation

In this section, we perform MEDR, FAD, direct transmission and the epidemic routing protocol in NS-2.33, and compare the performance of the four protocols from the following points of view: data delivery ratio, data delivery delay, and network lifetime. In addition, we also analyze the impacts of different experimental parameters on the protocols.

We assume the data generation of each sensor follows a Poisson process with an average arrival interval from 10 s to 100 s. The simulation parameters and their default values are summarized in Table 1. Specifically, in the MEDR, FAD and epidemic protocols, each sensor broadcasts a hello message to all its neighbors every 0.5 s, which is essential for mutual collaboration among sensors. In direct transmission, each sensor just communicates with the sink node directly, so only the sink node needs to broadcast hello messages periodically.

### 4.1. Impact of Message Generation Ratio

**Figure 4.**
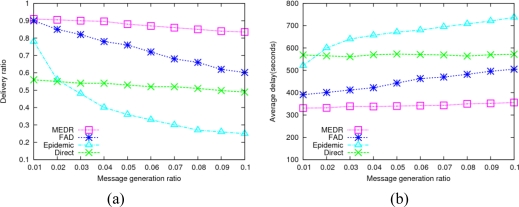
Impact of message generation ratio. **(a)** Average delivery ratio; **(b)** Average delay.

In the simulation, we vary the data generation rate in order to evaluate the performance of the four protocols under different transmission loads. As the date generation rate varies from 0.01 to 0.1 message/s, the performance of four protocols is as shown in Figure 4.

From Figure 4(a) we can see that MEDR achieves the highest data delivery ratio, obviously outperforming the other three protocols, which means that MEDR provides a more efficient data gathering scheme for DTMSNs. The direct transmission has the lowest data delivery ratio, since sensors just communicate with the sink node directly in this protocol, and if a sensor has no chance to move into the communication range of the sink node, those data generated by it may never be delivered successfully. We also notice that the data delivery ratio of the epidemic protocol is higher than direct transmission when the data generation rate is very low, but the value decreases dramatically as the data generation rate increases. This is due to MAC layer collision and rapid exhaustion of the limited network resources resulting from forwarding a tremendous amount of copies in epidemic routing. In addition, we find that FAD outperforms direct transmission and epidemic protocol as to the data delivery ratio. As the transmission load increase, its performance descends gradually since generating very many copies in this protocol. What’s more, the performance of FAD is unstable, which is influenced by the timer expiration value Δ and parameter α greatly. To have a fair comparison, we adjust these two parameters to get the close-to-optimal performance.

### 4.2. Impact of Node Density

The connectivity of DTMSN is closely related to the density of sensor nodes. The following experiments show the network performance of the four protocols with different sensor node density.

**Figure 5.**
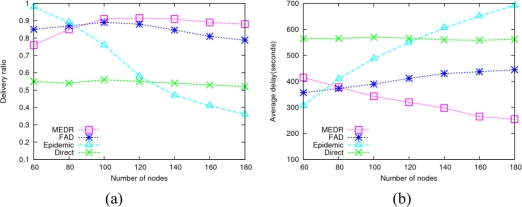
Impact of node density. **(a)** Average delivery ratio; **(b)** Average delay.

As shown in Figure 5(a), the epidemic protocol almost achieves the upper bound of the data delivery ratio when the node density is very low, since low node density means low transmission load and a small amount of wireless collisions. As the node density increases, the number of message copies increases dramatically in epidemic routing, which results in an increasing number of collisions and the reduction of the data delivery ratio. FAD shows slightly better data delivery ratio than MEDR when the node density is very low. This is due to poor connectivity resulting from very low node density, which influences the performance of MEDR. With the increment of node density, the connectivity of the network is enhanced, and thus the performance of MEDR improves rapidly. When the node number reaches 90, MEDR outperforms the other three protocols. As far as the performance of FAD is concerned, when the node density is very low, it’s difficult for a node to meet another node with higher delivery probability to help forward messages; when the node density is high, FAD will generate large numbers of message copies, which expends the limited resources of bandwidth and buffer quickly. Thus the performance of FAD descends under the two cases above. In direct transmission scheme, mobile sensors just communicate with the sink node directly, so the performance of this protocol has almost nothing to do with the node density.

### 4.3. Impact of Moving Speed

**Figure 6.**
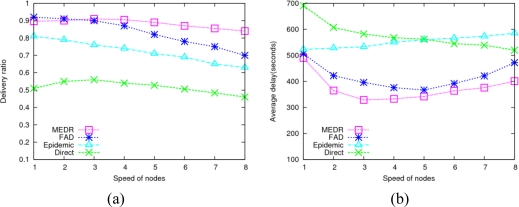
Impact of node move speed. **(a)** Average delivery ratio; **(b)** Average delay.

In DTMSNs, the moving speed of sensor nodes has a considerable impact on the performance of data gathering. The following experiments show the network performance of the four protocols under different node moving speeds. As shown in Figure 6, with the increment of node moving speed (from 1 m/s to 5 m/s), the performance of MEDR and direct transmission becomes better since higher moving speeds can shorten the time interval of meeting with the sink node, which means more delivery chances, so the performance is improved. However, once the moving speed exceeds 5 m/s, increasing the moving speed will decrease the performance of MEDR, FAD and direct transmission. This phenomenon can be explained by the following two aspects: on the one hand, due to the communication range of each node being fixed to 5 m in this paper, very fast moving speeds may make the connection time between any two nodes too short to complete the delivery of messages when they meet each other; on the other hand, the period of sending a hello message is fixed to 0.5 s for each node. If a sensor moves too fast (e.g., 8 m/s), the sensor node has already moved 4 m during the time interval of sending two hello messages, which is prone to result in the updating of neighbor list hardly keeping up with the changes of the network topology. In Figure 6, we also find that the performance of the epidemic protocol descends with the increase of node moving speed. The reason is that nodes meet more frequently at higher moving speeds, which results in tremendous amounts of message copies being generated and forwarded among meeting nodes, as well as a mass of wireless collisions, correspondingly.

### 4.4. Network Life

The network life is an important assessment criterion of a protocol from the aspect of total energy consumption. The experiments show the network lifetime of the four protocols, and the results are described in Table 2. We assume that the energy of the sink node is unlimited, and the initial energy of each sensor is 10 J. The energy needed in each transmission and receiving action is as specified in paper [27]. We consider the network dead when over a half of all sensor nodes deplete their energy.

We can see from Table 2 that the direct transmission protocol achieves the longest network lifetime, since sensors does not receive or transmit any messages except those generated by the sensor itself, and thus much energy can be saved. Sending and receiving too many messages copies expends too much energy in epidemic routing, so its network lifetime is the shortest among the four protocols. Moreover, we also see that MEDR has much longer network life than FAD. The reason is that, different from the multiple-copy feature of FAD, MEDR is a single-copy transmission scheme, thus it can efficiently reduce communication overhead. In a word, the total energy consumption of MEDR is much less than FAD and epidemic routing, which demonstrates the advantage of our proposed protocol in the aspect of economizing energy.

What deserves to be mentioned is it is almost equal that the energy consumed for sending and receiving hello messages in the MEDR, FAD and epidemic protocols, respectively. However, it is obviously different that the proportion of the energy expended on hello messages accounts for the total energy consumption in each one of the above three protocols. Table 3 shows the network life without considering the energy consumed by hello messages. From it we see that the network lifetime of MEDR reaches 2,884.7 hours, while the network lifetimes of FAD and epidemic are 196.18 and 27.16 hours respectively. Therefore, without considering the energy consumed by hello messages, MEDR clearly outperforms FAD and epidemic from the view of energy savings.

### 4.5. Impact of the parameter t_hop_

**Figure 7.**
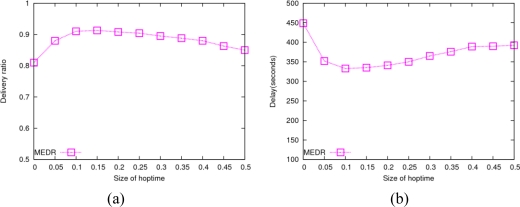
Impact of *t_hop_* in MEDR. **(a)** Average delivery ratio; **(b)** Average delay.

As is described in Section 4.3, the constant parameter *t_hop_* represents the estimated time used to transmit a message to a neighbor node. This parameter is employed to compute the MED and its validity period, so it has a certain impact on the routing performance of the MEDR protocol. Figure 7 shows the impact of *t_hop_* on the data delivery ratio and average delay in MEDR routing as it varies from 0 to 0.5 s. We see from this figure that the performance of MEDR descends when the value of *t_hop_* is too small or too big. The reason is that the validity period of MED is very short when the value of *t_hop_* is too small, which causes some invalid transmissions; when the value of *t_hop_* is too big, some paths which should be used for messages transmission are omitted. Moreover, MEDR achieves a good routing performance with a stable data delivery ratio and average delay, while the value of *t_hop_* is within the range from 0.1 s to 0.25 s.

## 5. Conclusions

In this paper, we propose a novel routing protocol called MEDR tailored for DTMSNs. In MEDR, each sensor maintains two important parameters: the minimum expected delay (MED) and its valid period. According to the above parameters, MEDR can efficiently find out and utilize the temporary and local multiple-hop connected paths which are dynamically formed by moving nodes to improve the performance of data gathering. We evaluate the performance of MEDR, direct transmission, epidemic and FAD algorithms by extensive simulations. The experimental results show that our proposed MEDR protocol outperforms the other three approaches in terms of message delivery ratio and average delivery delay, and its transmission overhead is much less than that of FAD and epidemic routing.

## Figures and Tables

**Table 1.** Simulation parameters.

**Parameter**	**Default value**
Network size (m)	200 × 200
Number of sensor node	100
Transmission radii *r* (m)	5
Speed of sensor node v (m/s)	3
Maximum queue size of sensor (message)	200
Data message size (bytes)	100
Control message size (bytes)	25
Message generation ratio (message/s)	0.01
Maximum delay tolerant value (s)	1,800
Position of sink node	(100, 100)
*t_hop_* value(s)	0.1

**Table 2.** Network life with default parameters.

	**MEDR**	**FAD**	**Epidemic**	**Direct Transmission**
Network Lifetime (hours)	108.4	71.8	22.73	4,675.12

**Table 3.** Network life without the energy consumed by hello messages.

	**MEDR**	**FAD**	**Epidemic**	**Direct Transmission**
Network Lifetime (hours)	2884.7	196.2	27.16	4978.23
